# P-1948. Mortality and Safety Outcomes of Isavuconazole in Patients with Invasive Aspergillosis and Impaired Liver Function: A SECURE Trial Emulation Approach

**DOI:** 10.1093/ofid/ofaf695.2116

**Published:** 2026-01-11

**Authors:** Ming-Ying Ai, Wei-Lun Chang, Ming-Shyan Wang

**Affiliations:** Far-Eastern Memorial Hospital, New Taipei, Taipei, Taiwan (Republic of China); Far-Eastern Memorial Hospital, New Taipei, Taipei, Taiwan (Republic of China); Far-Eastern Memorial Hospital, New Taipei, Taipei, Taiwan (Republic of China)

## Abstract

**Background:**

Invasive aspergillosis poses a major challenge, especially in patients with impaired liver function, where treatment options are limited due to hepatotoxicity concerns. Isavuconazole, an antifungal agent with a favorable hepatic safety profile compared to voriconazole, is increasingly used in this high-risk group. However, the SECURE trial excluded patients with liver impairment, leaving the safety and outcomes of isavuconazole unknown in this population. Using a trial emulation framework based on the SECURE concept, this study aims to estimate the causal effect of isavuconazole on mortality and liver-related adverse outcomes in patients with liver dysfunction over a five-year hospital-based observation.

**Figure:**
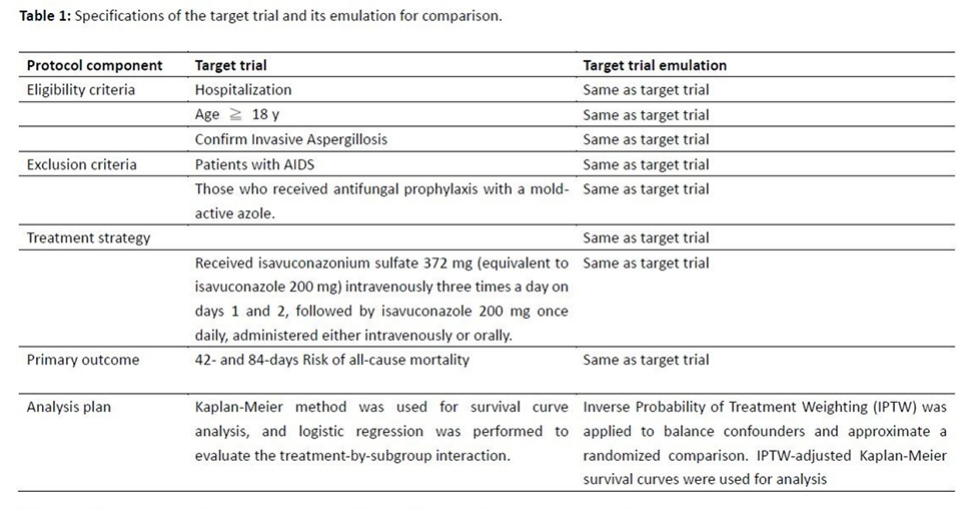

**Figure:**
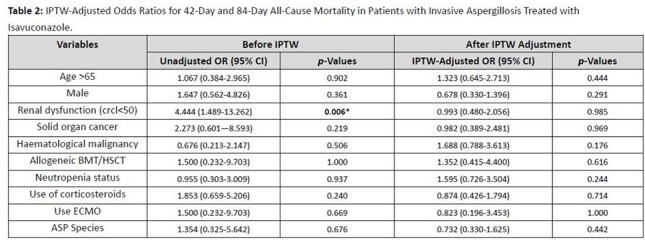

**Methods:**

This retrospective cohort study emulated a hypothetical randomized trial by defining eligibility criteria, treatment assignment, and follow-up strategies. Data were collected from hospitalized patients receiving isavuconazole for invasive aspergillosis between 2019 and 2024. Patients were stratified into liver dysfunction (bilirubin ≥3×ULN, ALT/AST ≥3×ULN, cirrhosis, or hepatic failure) and normal liver function groups. Inverse Probability of Treatment Weighting (IPTW) balanced confounders. Kaplan-Meier survival curves compared 42-day and 84-day mortality, and logistic regression assessed liver-related safety outcomes. A *p*-value < 0.05 was considered significant.

**Figure:**
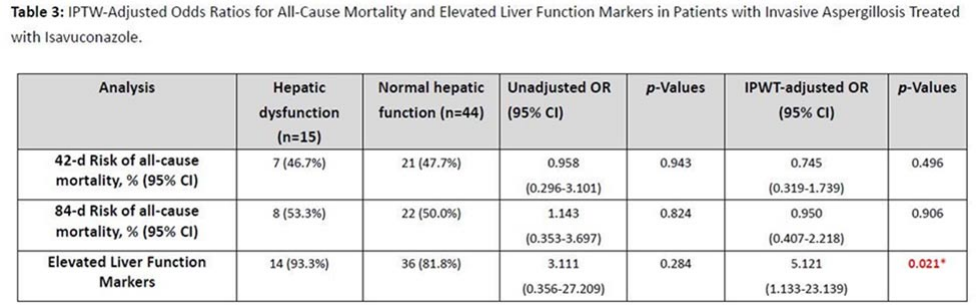

**Figure:**
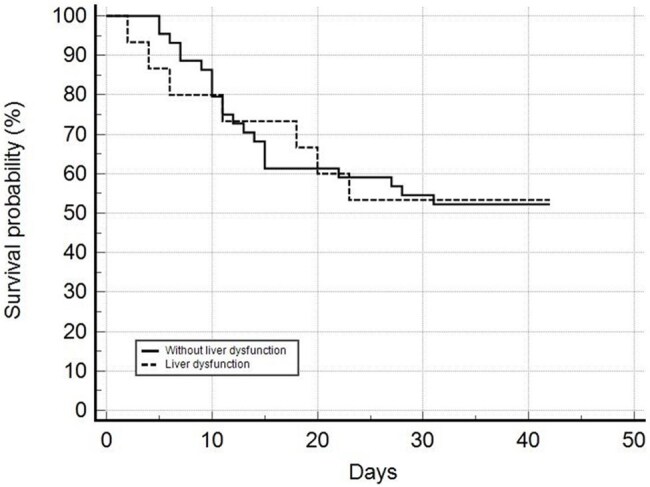

**Results:**

Fifty-nine patients were included. After IPTW adjustment, there was no significant difference in 42-day (IPTW-adjusted OR: 0.745, 95% CI: 0.319–1.739, p=0.496) or 84-day mortality (IPTW-adjusted OR: 0.950, 95% CI: 0.407–2.218, p=0.906) between groups. However, patients with liver dysfunction had higher odds of elevated liver markers (IPTW-adjusted OR: 5.121, 95% CI: 1.133–23.139, p=0.021). Increased hepatic impairment was not associated with higher mortality (p=0.517).

**Conclusion:**

This trial emulation suggests that isavuconazole can be safely used in patients with liver dysfunction without significantly impacting mortality. Although elevated liver markers were more common, they did not translate into worse survival outcomes, supporting the hepatic safety of isavuconazole in this vulnerable group.

**Disclosures:**

All Authors: No reported disclosures

